# Dickson polynomial-based secure group authentication scheme for Internet of Things

**DOI:** 10.1038/s41598-024-55044-2

**Published:** 2024-02-28

**Authors:** Salman Ali Syed, Selvakumar Manickam, Mueen Uddin, Hamed Alsufyani, Mohammad Shorfuzzaman, Shitharth Selvarajan, Gouse Baig Mohammed

**Affiliations:** 1 Department of Computer Science, Applied College Tabarjal, Jouf University, Sakaka, Al-Jouf Province Kingdom of Saudi Arabia; 2 National Advanced IPv6 Centre (NAv6), Universiti Sains Malaysia, 11800 Gelugor, Penang Malaysia; 3 College of Computing and IT, University of Doha for Science and Technology, 24449 Doha, Qatar; 4 Department of Computer Science, College of Computing and Informatics, Saudi Electronic University, 11673 Riyadh, Kingdom of Saudi Arabia; 5 Department of Computer Science, College of Computers and Information Technology, Taif University, 21944 Taif, Kingdom of Saudi Arabia; 6 School of Built Environment, Engineering and Computing, Leeds Beckett University, Leeds, LS1 3HE UK; 7 Department of Computer Science and Engineering, Kebri Dehar University, 250 Kebri Dehar, Ethiopia; 8 Department of Computer Science and Engineering, Vardhaman College of Engineering, Hyderabad, India

**Keywords:** Conditional privacy preservation, Certificate-less, Group authentication scheme, Internet of Things, Dickson polynomial, Blockchain technology, Engineering, Electrical and electronic engineering

## Abstract

Internet of Things (IoT) paves the way for the modern smart industrial applications and cities. Trusted Authority acts as a sole control in monitoring and maintaining the communications between the IoT devices and the infrastructure. The communication between the IoT devices happens from one trusted entity of an area to the other by way of generating security certificates. Establishing trust by way of generating security certificates for the IoT devices in a smart city application can be of high cost and expensive. In order to facilitate this, a secure group authentication scheme that creates trust amongst a group of IoT devices owned by several entities has been proposed. The majority of proposed authentication techniques are made for individual device authentication and are also utilized for group authentication; nevertheless, a unique solution for group authentication is the Dickson polynomial based secure group authentication scheme. The secret keys used in our proposed authentication technique are generated using the Dickson polynomial, which enables the group to authenticate without generating an excessive amount of network traffic overhead. IoT devices' group authentication has made use of the Dickson polynomial. Blockchain technology is employed to enable secure, efficient, and fast data transfer among the unique IoT devices of each group deployed at different places. Also, the proposed secure group authentication scheme developed based on Dickson polynomials is resistant to replay, man-in-the-middle, tampering, side channel and signature forgeries, impersonation, and ephemeral key secret leakage attacks. In order to accomplish this, we have implemented a hardware-based physically unclonable function. Implementation has been carried using python language and deployed and tested on Blockchain using Ethereum Goerli’s Testnet framework. Performance analysis has been carried out by choosing various benchmarks and found that the proposed framework outperforms its counterparts through various metrics. Different parameters are also utilized to assess the performance of the proposed blockchain framework and shows that it has better performance in terms of computation, communication, storage and latency.

## Introduction

Smart city is one of the prominent applications of Internet of Things (IoT)^1^. It integrates a coercion of various capabilities such as omnipresent sensing, diverse network infrastructures and sophisticated information processing and control systems^2^. The main aim of a smart city is to provide an eco-friendly habitat by enhancing the socio-economic quality, increased sustainability, smart transportation and convenience. IoT provides a wide variety of applications relating to smart energy, smart grid management, smart surveillance and transportation systems, smart socio-economic and cultural community, smart waste management, smart healthcare, and so on. In case of Industries, IoT paves the way for efficient applications pertaining to inventory and asset control, remote monitoring, supply chain and logistics, smart production and manufacturing and others^3^. Smart city applications raise a serious of concerns and challenges in terms of security and privacy threats ranging from unauthorized intrusions, acknowledgements, disruption, mitigation, scrutiny and devastation^4^. Internet of Things also raises security concerns comprising of data identification, data authentication, data integrity, availability, confidentiality, access privileges, data privacy and trust^5^. The information transmitted between the IoT devices happens through a wide open communication channel they are highly prone to security and privacy attacks^6^. It is obvious that intruders may create bogus messages to exploit details relating to services, diagnosis and thereby control by altering the original messages. It is apparent that the malevolent intruders might perform denial-of-service attacks thereby disturbing and maneuvering the services^7^. Information pertaining to an individual might be hacked and traced thereby causing damages either physically or by virtual means thereby threatening the privacy^8^. Sometimes traveling route of a vehicle when falls into the hands of intruders they may alter or destroy or deliver wrong or false messages which may lead to unfavorable situations. In such a case anonymous communications is highly essential between the IoT devices of one group to the other^9^.

The identities of IoT devices are at high risk which enables the malevolent attackers to identify the location leading to location privacy. Traceability is one of the additional problems that Internet of Things is prone to^10^. The architecture of IoT addresses various problems pertaining to network, intrusion, middleware, application but it fails to address concern relating to trust and identity preservation. Several of research works concerning security and privacy schemes for IoT devices are highly dependent on a single central entity called trusted authority (TA). Hence in order to perform the transmissions of messages the trusted authority of one set of IoT device in an area has to perform a trusted handshake with the other by the exchange of secret keys which gets installed inside the devices deployed. Therefore it is possible to attack the IoT devices installed in an environment in order to gain malicious access by means of side channel and tampering attacks. Upon any unfavorable situations, it is essential to revoke malicious entity inside the Internet of Things network which is a sole responsibility of the trusted authority. The number of messages to be exchanged is to be verified at the receiving end which poses a computational overhead leading to signature forgery, side channel and denial-of-service attacks^11^. Since the IoT network involves a huge traffic generated by a wide variety of infrastructure; it is essential to design an authentication scheme that supports heterogeneity of devices^12^. Hence designing an efficient authentication scheme for IoT network must ensure the following objectives like privacy preservation, traceability, tamper-proof protection, secure and lightweight. In order to mention the aforementioned security and privacy concerns; blockchain technology provides a viable solution^13,14^. Blockchain is a decentralized, traceable, explicit and immutable ledger that contains records of transactions in peer-peer networks (P2P)^15^. Bitcoin a viable solution offered by blockchain to facilitate digital payments among the two entities thereby alleviating the use of a central authority^16^. Each activity inside the IoT network gets recorded as a transaction and is stored in a blockchain. Every complicated legalized course of action and data transmission can be done with the help of smart contracts. Due to the utilization of smart contracts and dispersed applications, an immense sovereignty and free-will can be gained for each individual transaction in an IoT network. Blockchain exhibits a wide variety of characteristics namely absolute authentication, security, privacy, efficient deployment and maintenance^17^. In order to alleviate the tampering attacks on IoT devices, it is essential to include hardware security using physical unclonable functions (PUFs)^18^.

### Motivation

Due to these characteristics, a group authentication scheme has been proposed to perform the authentication amongst a group of IoT devices managed by different trusted authority has been proposed. In order to facilitate this, Dickson polynomials over a finite field can be utilized for public key cryptosystems^19^. The inherent nature of semigroup property and permutation behaviors exhibited by the Dickson polynomials makes it suitable to design an authentication scheme. This semigroup property is utilized in the proposed group authentication scheme in order to achieve authentication amongst the IoT devices. Smart contracts of blockchain are used to ensure a secure communication with distinct trusted authorities in a disseminated environment thereby facilitating Dickson polynomial-based group authentication scheme. The design helps in assuring a unified base for associating the trusted authorities of distinct trusted authorities to have a secure communication. With the help of this several use cases can be built benefitting the smart city and Industrial IoT.

### Our contributions

The proposed Dickson polynomial based secure group authentication scheme for Internet of Things involves the following contributions:

- Most of the proposed authentication schemes are designed for the authentication of individual devices and the same is used for group authentication whereas the proposed Dickson polynomial based secure group authentication scheme is proposed peculiar for performing group authentication.
- In our proposed authentication scheme, the Dickson polynomial is used to generate the secret keys allowing the group to get authenticated without creating excessive network traffic overhead. Dickson polynomial has been utilized for the for the group authentication of IoT devices.
- In order to ensure the secure, efficient and fast message transmission among the distinct IoT devices of each group deployed at various locations are facilitated by the utilization of blockchain technology.
- In our proposed Dickson polynomial based secure group authentication scheme, physically unclonable hardware identification is used thereby being resistant towards tampering, side channel and signature forgery, replay, man-in-the middle, impersonation and ephemeral key secret leakage attacks.
- The proposed scheme has been implemented by using a hardware based physically unclonable function.

### Organization of the paper

The paper has been organized as follows: “Introduction” section describes the introduction; “Related works” section deliberates the related work for our proposed system; “System preliminaries” section specifies the system preliminaries; “Proposed group authentication framework” section describes the proposed group authentication scheme; “Security analysis” section provides details about the security analysis; “Performance analysis” section deliberates details on the performance aspects of the proposed scheme and “Conclusion” section concludes the paper.

## Related works

Internet of Things comprises of devices used to capture and transfer data when deployed in remote environments^20^. IoT devices are usually placed in unprotected environments and are highly susceptible to security issues namely signature forgery, illegal access, physical cloning, side channel, eavesdropping, malicious node injection, man-in-the-middle and distributed denial of service attacks^21^. An estimate from Kaspersky states that in 2021 summer nearly 1.5 billion IoT devices are compromised by using a simple remote access protocol called Telnet. Slack communications, weak passwords and loose authentications are the most common security breaches faced by IoT devices. Various authors have proposed have proposed distinct authentication methods to address the problem of security in IoT. This section provides details on various literatures proposed by various researchers where it has been segregated based on the Schemes based on generic authentication of IoT devices, Schemes based on group authentication and Authentication schemes utilizing blockchain technology.

### Schemes base on generic authentixcation

Shah et al.^22^ proposed an authentication mechanism for IoT devices and IoT servers using secure vaults. Multi-key based Mutual authentication mechanisms have been utilized for the proposed scheme. In this scheme, message transmission happens by exchanging the shared secret among the IoT device and the server by means of equal key size which is called as secure vault. However the keys of equal size become a crucial problem that the scheme faces which leads to offline password guessing attacks or cloning attacks. Wallrabenstein^23^ proposed a physically unclonable function based elliptic curve cryptography authentication scheme for Internet of Things. The approach has been combined with elliptic curve cryptography which substantially reduced the computational and storage overhead incurred during authentication. The PuF based schemes provides an alternative security means against tampering attacks it suffers from side channel attacks. Aman et al.^24^ comes up with a PUF based two-factor authentication scheme for Internet of Things environment. The scheme utilized wireless signal features that acts as an effective tool for securing IoT devices from spoofing, cloning, tampering and other attacks. Though the scheme efficiently addresses locating the compromised or the breached IoT devices it suffers from tampering and side channel attacks. Gope et al.^25^ proposed a lightweight and privacy preserving two factor authentication scheme for IoT devices. The scheme lacks its efficiency in case of offline password guessing attacks. Goswami et al.^26^ came up with a universal subscriber identity module (USIM) based remote registration and group authentication for 5G networks. The authentication followed is a group authenticated key agreement protocol is utilized (5G-AKA). If and when the number of IoT device load gets increased there will be a computational burden. Yadav et al.^27^ proposed a lightweight extensible authentication protocol (EAP) designed for Internet of Things environment. The scheme suffers computation overhead across unidentified attacks. Patel et al.^28^ came up with an authentication entity can be used in the smart city between the IoT sensors and the receivers. The authenticating entity is highly responsible for providing authentication between the IoT devices prior to data transmission. Though the authentication scheme is an inter-device authentication scheme it utilizes elliptic curve cryptography. Sharma et al.^29^ came up with a set of primitives and a cubic equation by introducing a secure element in between the IoT device and the receiver. Though the method becomes efficient it involves complex mathematical operations which needs to be made to robust for high speed wireless environment.

### Scheme based on group authentication

Albeshri et al.^30^ proposed a trusted authority based lightweight authentication scheme. The authentication scheme utilized elliptic curve cryptography. The scheme has been proposed by introducing a security element specifically to perform authentication in case of Industrial Internet of Things environment. Their scheme addresses the problem of routing when an IoT device wants to join in a group by distracting it. The authentication scheme utilizes image based hashing technique for authentication However; the proposed scheme suffers from man-in-the-middle attacks. Mahelle et al.^31^ proposed a group authentication mechanism that utilizes image hashing crosschecking before joining any group. Threshold based cryptography approach is used for address the authentication of IoT nodes. Their scheme addressed the problem of battery exhaustion attack. El Mouaatamid et al.^32^ proposed a scalable group authentication scheme based on combinatorial design to address fault tolerance in IoT. Their scheme partially addresses the objective by adjusting with the faults. Aydin et al.^33^ came up with a lightweight and a flexible group authentication scheme to reduce the energy consumption. Their scheme shows resistance towards replay and man-in-the-middle attacks. However the approach suffers from side channel and physical cloning attacks. Yildiz et al.^34^ proposed a physically unclonable function based lightweight group authentication and key distribution protocol by utilizing factorial tree and Chinese remainder theorem.

### Schemes based on blockchain technology

Gong et al.^35^ discussed the use of blockchain technology in IoT device authentication mechanism. The identity theft or breach can be prevented by storing the information in blockchain to facilitate IoT device authentication utilizing distributed ledger. Ferreira et al.^36^ designed a scheme where the API gateway is designed that can be used by the IoT devices and the network gateway where signing, identification and authorization of messages can be facilitated. The proposed methodology has to be tested under real-time scenarios. The problem of distributed denial of service attack has been alleviated by building a guard shield using the block chain smart contracts. Jia et al.^37^ came up with an identity based cross-domain authentication scheme for Internet of Things by using blockchain technology. Authentication to smart dust IoT systems are difficult to achieve due to the increase in the volume of the devices which increases the time required for authentication. Park et al.^38^ came up with a solution based on blockchain by reorganizing the original block structure of the blockchain to a binary tree blockchain. This provides an effective increase in the authentication time of about 10%. However the reorganization still suffers from additional overhead. Honar et al.^39^ introduced the concept of clustering for IoT device authentication utilizing blockchain. In this technique the IoT devices are grouped locally into a cluster and a cluster head will be elected which facilitates the process of authentication for its group members. The proposed work achieves the objective of providing security and privacy it has to be tested for real-world scenarios. Tahir et al.^40^ proposed a novel authentication and authorization framework for blockchain enabled IoT networks using probabilistic models. The scheme also utilized random numbers for authentication through joint conditional probability. The major drawback is that the computation overhead increases with increase in the number of IoT devices and data. Latif et al.^41^ proposed new blockchain architecture for secure and trustworthy operations in case of Industrial IoT. The proposed scheme utilized asymmetric cryptographic operations. However the device level authentication is difficult to be achieved. Mehbodniya et al.^42^ came up with a scalable and energy efficient proof-of-authentication consensus algorithm. The algorithm utilized modified lamport-merkle digital signature for signature verification and generation medical internet of things. The scheme achieved faster security.

From the literature survey it is apparent that group authentication based on a decentralized approach is very limited and are not enough. Existing schemes on group authentication are proposed for centralized group authentication of a group of IoT devices. Therefore there is no framework addressing the decentralized group authentication when the IoT devices are under the governance of distinct entities. Our proposed scheme aims to present a secure group authentication scheme when the group of IoT devices under distinct trusted authority wants to communicate which are situated at remote locations.

## System preliminaries

This section provides the relevant technology, methods used, system model, design goals and adversary model utilized for our Dickson polynomial based secure group authentication scheme for Internet of Things.

### Blockchain

**Figure 1:**
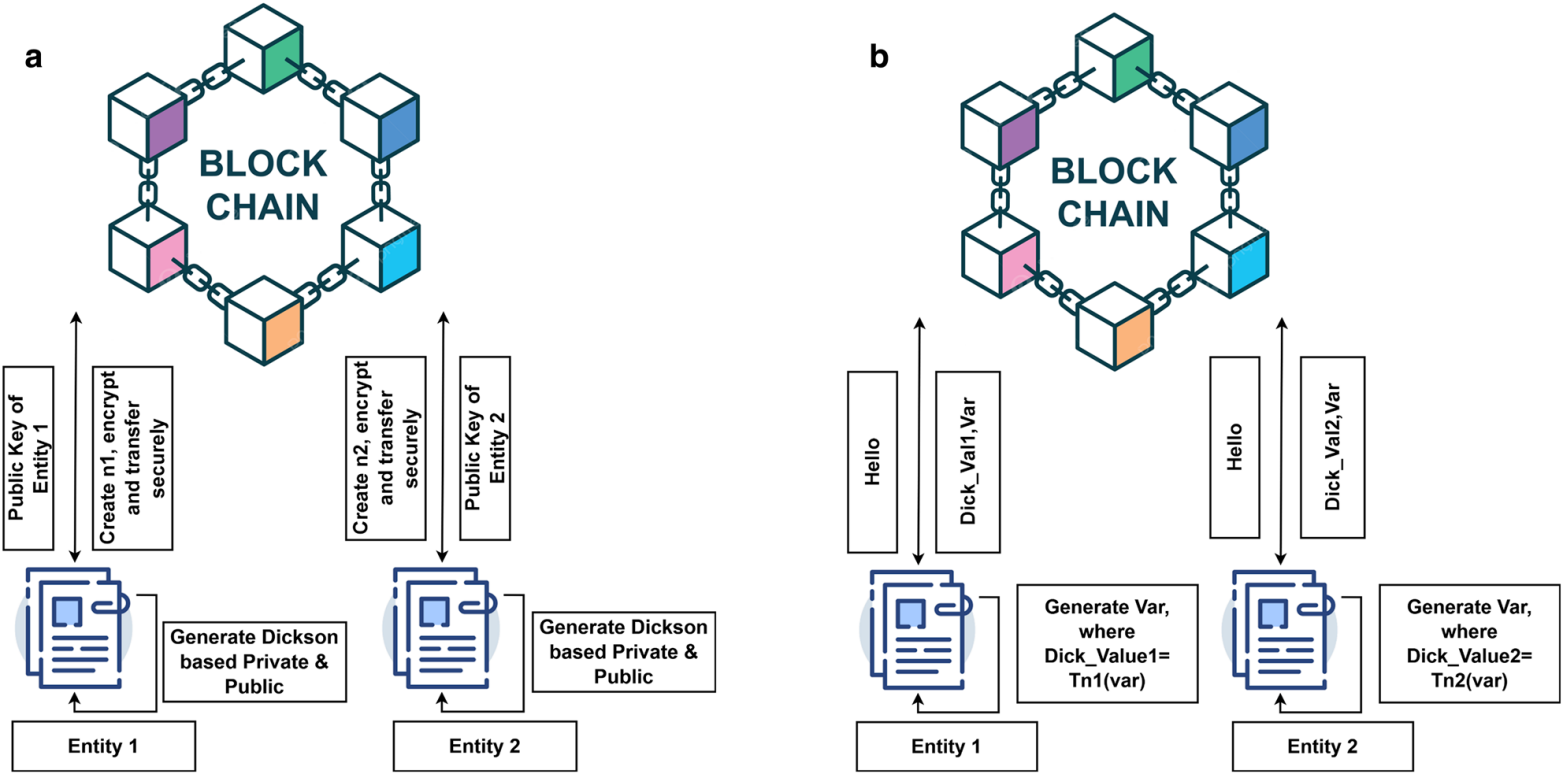
Working methodology of the Dickson polynomial for group authentication (**a**) Dickson polynomial is shared secretly with all entities. Working methodology of the Dickson polynomial for group authentication (**b**) Verification of group members.

The major advantage of utilizing blockchain technology is its ability to alleviate the central governance. The transactions are executed in a decentralized manner where the consensus procedure is utilized to authenticate the trustworthiness in each node. This provides the capability for the transactional data to be lucid, irreversible, provable, authentic, tamper-proof and retractable. In our proposed scheme a open-source blockchain called Ethereum is utilized^43^. Ethereum blockchain possess an in-built turing machine which allows the execution of transactions^44^. The transactions are built on state-transition functions. Gas is a unique pricing currency utilized in the Ethereum blockchain to facilitate resource allocation in proportion to the amount of transactions processed and spam protection^45^. The proof of Work algorithm is EThash utilized by the Ethereum mining nodes^46^. Keccak-256 a modified variant of SHA3 is the hashing algorithm used in Ethereum^4,47–49^. Figure 1 depicts the basic structure of the blockchain.

### Physically unclonable function (PUF)

Physically Unclonable Function (PUF) is a semiconductor based electronic material utilizing micro components. It generates an uncertain output O when it gets elicited by means of a input I. The input–output pairs correspond to a number of pairs of inputs $I_{i}$ and their analogous outputs $O_{i}$^50^. They deliver a distinct output which is proportional to that of its input and the physical shape. Therefore, when an intruder mess with this device the physical shape of it gets altered this certainly results in a change in the output. This property of physically unclonable function makes it superior to be deployed at a far way distance.

### Dickson polynomial

Dickson polynomial was first proposed by Dickson^51^. The unique property of computational discrete hardness of a dickson polynomial has attracted researchers to propose a public key encryption scheme. Dickson polynomial finds its applications in number theory and cryptography. Let n be a non-negative integer and α $\in$ ${\text{F}}_{{\text{q}}}$; then the Dickson polynomial can be given by ${\text{D}}_{{\text{n}}} ({\text{x}},{\upalpha }$) over a finite field can be defined by the Eq. (1).

1 $${\text{D}}_{{\text{n}}} ({\text{x}},{\alpha }){\text{ }} = \mathop \sum \limits_{{i = 0}}^{{\left\lfloor {\frac{n}{2}} \right\rfloor }} \frac{n}{{n - i}}~\left( {\begin{array}{*{20}c} {n - i} \\ i \\ \end{array} } \right)( - \alpha )^{i} x^{{n - 2i}}$$

where $\left\lfloor {\frac{n}{2}} \right\rfloor$ is the largest integer $\ge \frac{n}{2}$. Dickson polynomial also gratified by using the recurrence relation such that ${\text{D}}_{{\text{n}}} ({\text{x}},{\upalpha }$) = x ${\text{D}}_{{{\text{n}} - 1}} ({\text{x}},{\upalpha }$)—${\alpha D}_{{{\text{n}} - 2}} ({\text{x}},{\upalpha }$) ; n $\ge$ 2 under the initial condition ${\text{D}}_{0} ({\text{x}},{\upalpha }$) = 2 and ${\text{D}}_{1} ({\text{x}},{\upalpha }$) = x and some of the other polynomials are as follows:

2 $${\text{D}}_{2} ({\text{x}},\upalpha ) \, = x^{2} - { 2 }\upalpha$$

3 $${\text{D}}_{3} ({\text{x}},\upalpha ) \, = x^{3} - { 3 }\upalpha$$

4 $${\text{D}}_{4} ({\text{x}},\upalpha ) \, = x^{4} - { 4}\upalpha x^{2} + { 2}\upalpha ^{2}$$

5 $${\text{D}}_{5} ({\text{x}},\upalpha ) \, = x^{5} - { 4 }\,5\upalpha x^{3} + { 5}\upalpha ^{2} x$$

The commutative property under composite operation is one of the classifying feature of a Dickson polynomial when $\upalpha$ = 0 or 1. Therefore the Dickson polynomial satisfies the semigroup property under composition that can be defined as

6 $$\begin{aligned} {\text{D}}_{mn} ({\text{x}},1) & = {\text{D}}_{m} ({\text{D}}_{n} \left( {{\text{x}},1} \right),1) \\ & = {\text{D}}_{m} \left( {x,1} \right) o {\text{D}}_{n} \left( {{\text{x}},1} \right) \\ & = {\text{D}}_{n} \left( {{\text{x}},1} \right) {\text{oD}}_{m} \left( {x,1} \right) \\ & {\text{ = D}}_{n} ({\text{D}}_{m} \left( {x,1} \right), 1) \\ & = {\text{D}}_{nm} ({\text{x}},1) \\ \end{aligned}$$

Though the Dickson polynomial finds a wide variety of properties the semigroup property is an essential property that can be utilized for the cryptographic applications. Let us consider two large integers p and q and another integer x, the semigroup property can be defined by the Eq. (7) as:

7 $${\text{D}}_{p} \left( {{\text{D}}_{q} \left( {\text{x}} \right) \left( {mod\,n} \right)} \right) = {\text{ D}}_{pq} \left( {\text{x}} \right)({\text{mod n)}}$$

Due to the inherent property of the semigroup; Dickson polynomial can be utilized to encrypt and decrypt the message m using Elgamal public key cryptography. In order to facilitate message transmission in a secure way,

Alice can produce a large integer denoted by ${\text{pka}}$ and another number x which calculates

8 $$Dick_{Alice} = D_{pka} \left( x \right)$$

where $Dick_{Alice}$ is the Dickson polynomial value for Alice. For Alice the public key can be denoted as (x, $Dick_{Alice}$) and pka is a private key. Let us consider that Bob has to send a message m to Alice. Bob creates a large integer pkb and calculates

9 $$Dick_{Bob} = D_{pkb} \left( x \right)$$

Then

10 $$D_{AliceBob} = D_{pkb} \left( {Dick_{Alice} } \right)$$

Bob calculates M as

11 $${\text{M }} = {\text{ m x}}D_{AliceBob}$$

Now bob sends an encrypted text Cwhich computes $D_{AliceBob}$ by using

12 $$D_{AliceBob} = D_{pka} \left( {Dick_{Bob} } \right)$$

Now Alice decrypts a message m by dividing the cipher text C with $D_{AliceBob}$. In our proposed group authentication scheme, Dickson polynomial based cryptosystem in combination with blockchain technology has been utilized for providing group based authentication to the IoT devices installed at various placements under distinct trusted authorities. Figure 1 provides the working methodology of our proposed scheme where the Dickson polynomial is used. The proposed authentication method has been segregated into two distinct phases. In the first phase, Dickson polynomial is used with a pre-determined degree for each object that belongs to a group of IoT device. A certain degree of Dickson polynomial is transmitted in a secure manner between two different entities by way of smart contracts which is depicted using Fig. 1a. In the second phase, group authentication is performed depicted by the Fig. 1b. The first group of IoT device calculates Dick_v1 using a degree n1 previously sent to it and a generated variable term1. Dick_v1 and term1 will be delivered to the smart contract by using the entity 1. The smart contract will transmit Dick_v1 to entity 2 after it has received these values. The entity 2 will use Dick_v1 and term2 to calculate Dick_v2 after receiving Dick_v1. Now the entity 2 will transmit Dick_v2 to the smart contract where it will compare Dick_v2 by computation which gets depicted in the figure to confirm the group membership. This process can be subsequently applied to more than two entities.

### Design goals

The main aim of our proposed secure group authentication scheme for Internet of Things is to facilitate efficient authentication and message exchange to provide security and privacy against the threat model. The design goals are listed as follow:

1. *Mutual Authentication* Any two groups of remotely located IoT devices must authenticate one another simultaneously.
2. *Shared secret Agreement* A shared secret has to be exchanged amongst any two entities after being authenticated.
3. *Message Integrity* Messages transmitted between two distinct group of IoT devices should not be changed while transmission.
4. *Anonymity* Only the receiver should have to decode the identity of the sender.
5. *Forward and Backward Secrecy* Exploitation of the secret key of an IoT device cannot be able to breach the transmission being secured by the previous shared secret or the next shared secret about to be exchanged.
6. *Resistance to Attacks* Our proposed scheme should be resilient to replay, man-in-the middle, impersonation, stolen smart card, ephemeral secret key leakage and offline password guessing attacks.

### Adversary model

The adversary model suggests that an adversary can perform eavesdropping and tampering attacks over the open wireless communication channel between the IoT device and the Trusted Authority. In addition they can forge the signature/identity of the group of an IoT network and pretend to belong to trick the trusted authority as a group member^52^. Canetti–Krawczyk (C–K) adversary has been considered to assess the security aspects of our proposed scheme^51^.

## Proposed group authentication framework

Our proposed group authentication framework coagulates the two different features namely smart contracts in blockchain technology and the semigroup property of the Dickson polynomial. These features enable us as an inspiration to design a group authentication scheme for internet of things environment.

### Scenario

**Figure 2:**
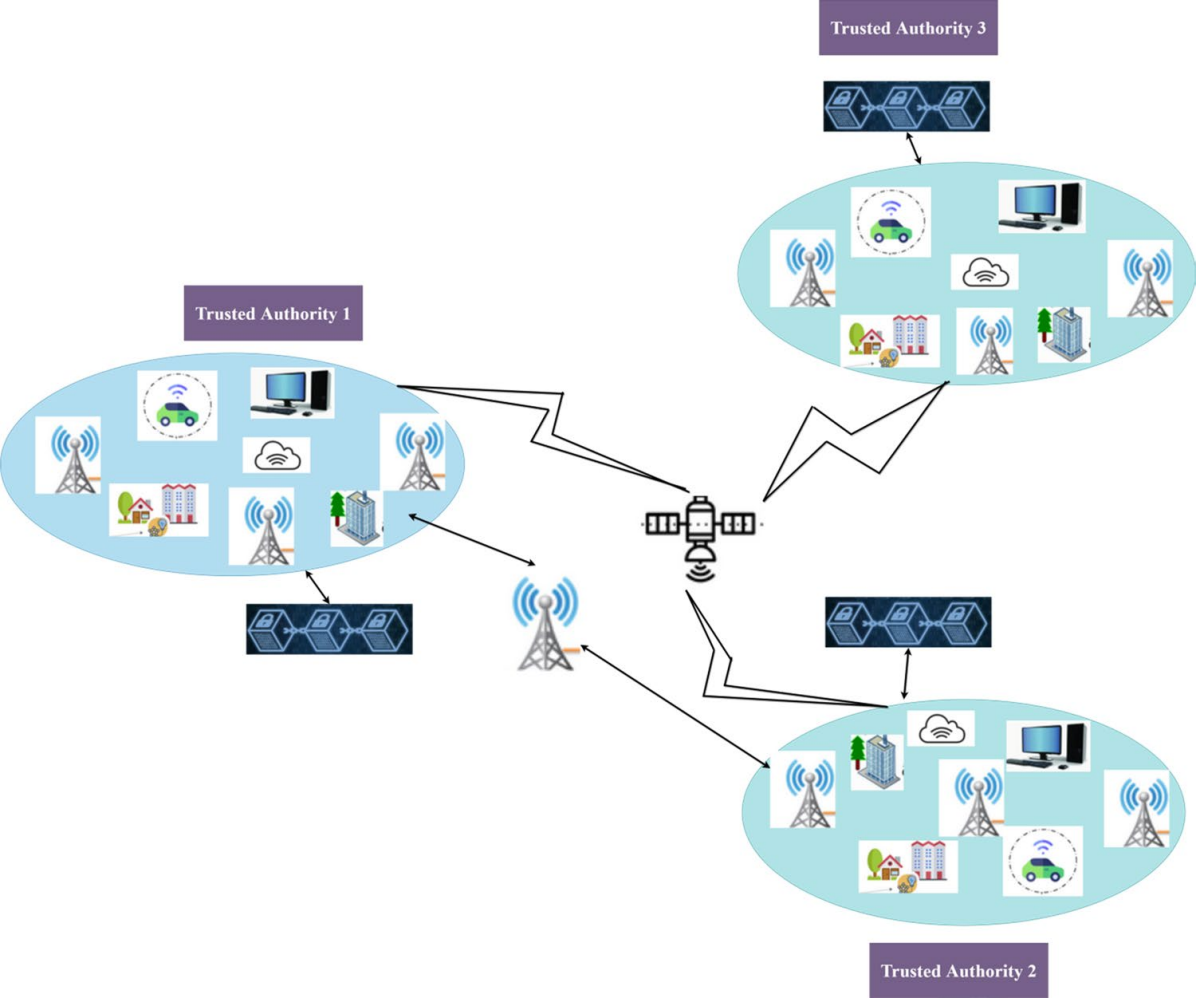
Scenario of Internet of Things.

**Figure 3:**
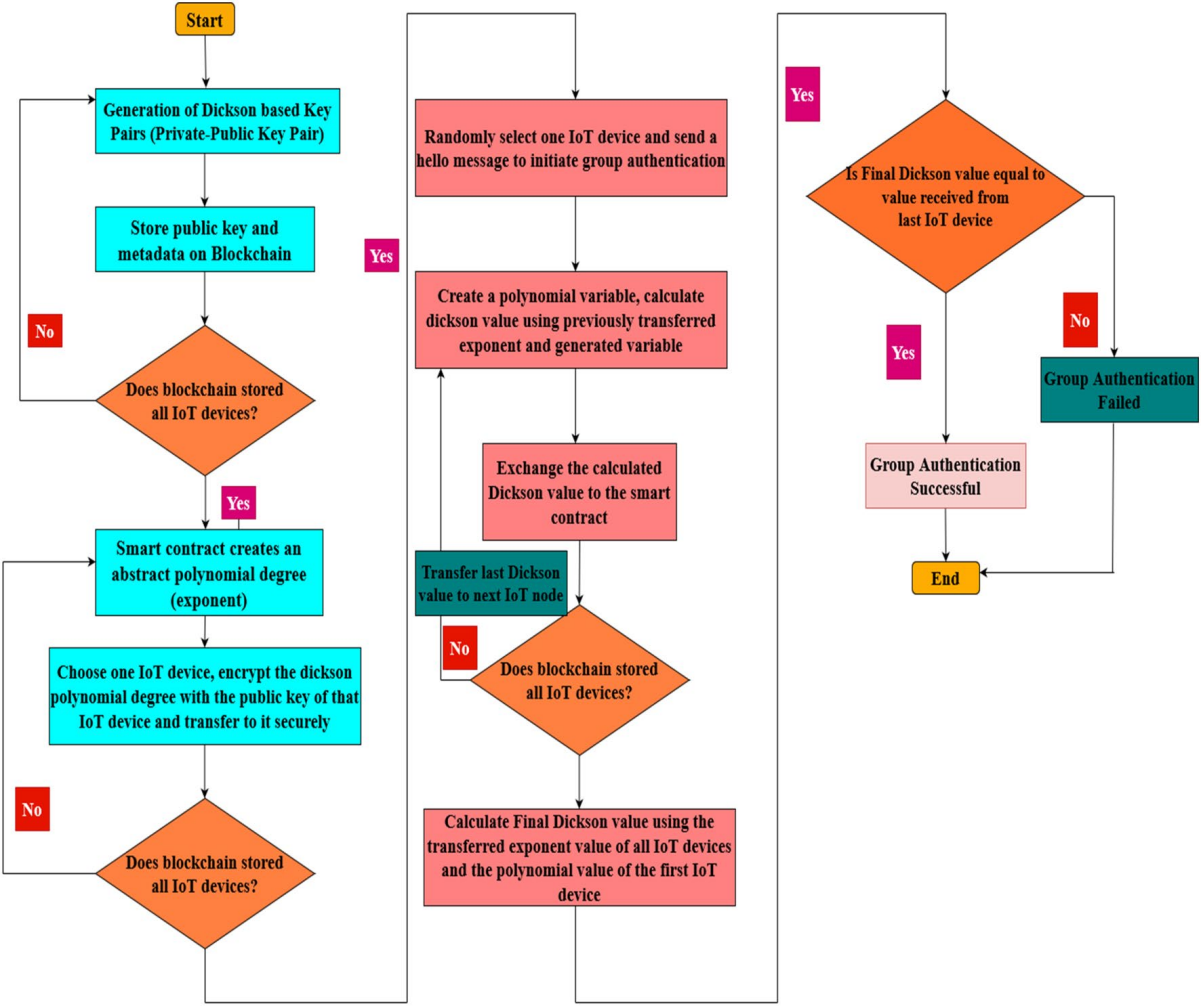
Authentication Mechanism of Dickson Polynomial based secure group Authentication Scheme for Internet of Things.

The authentication framework has been designed by keeping in mind when distinct smart cities or industries desire to communicate with the help of their IoT devices deployed at geographical locations. One such use case of a IoT network has been framed and depicted as shown in the Fig. 2 It is apparent that the framework has been designed by utilizing trusted authorities which are installed at various geographical locations. The trusted authority of a one particular IoT device group can perform the responsibilities pertaining to a smart city or industrial scenario. Each trusted authority is connected by various IoT devices installed in distinct premises. Group of the IoT device in an industrial area or a smart city constitute to report to a trusted authority. The trusted authority in a smart city gets connected to one blockchain node. Based on the configuration, requirements of the trusted authorities connected, the blockchain can be of different types namely shared blockchain, secret blockchain and communal blockchain. Each trusted authority comprises of various groups of IoT devices connected together in various cities or countries. When IoT devices of one group want to share data with the other the proposed authentication scheme provides efficient authentication. The authentication mechanism proposed involves various phases namely creation and storage of public key and metadata of each IoT device on the blockchain, secret sharing of Dickson polynomial degree or exponent via the blockchain to each IoT device; consequent creation of Dickson polynomial for each IoT device; and at the end finally comparing overall Dickson term with the last device’s Dickson term for efficient group authentication which are depicted using the flowchart shown in Fig. 3.

The group authentication framework functions in three phases namely creation phase and authentication phase. The phases of the proposed authentication scheme can be described as follows:

### IoT device registration phase

**Figure 4:**
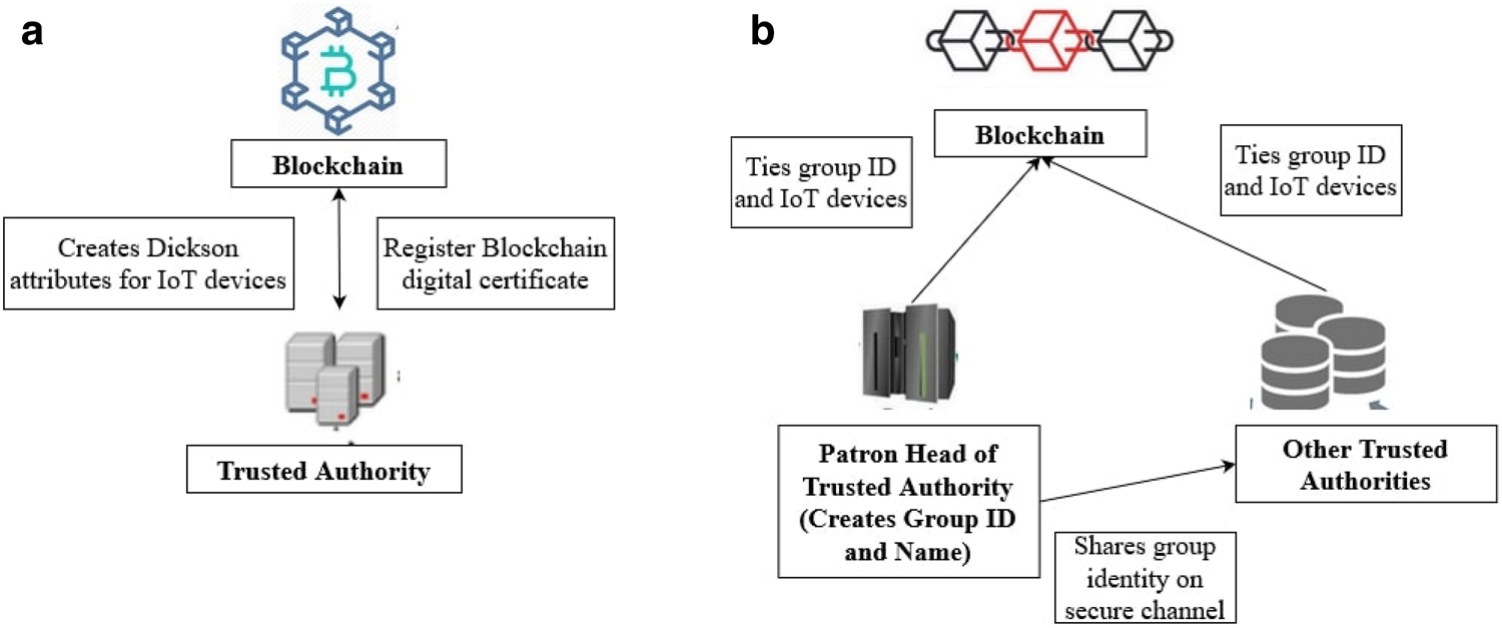
(**a**) IoT device Registration on Blockchain. (**b**) Creation of group and Tieing on Blockchain.

In this phase, all the trusted authorities register their corresponding IoT entities on the blockchain. IoT device registration and group creation architecture are given in Fig. 4a,b respectively. Each trusted authority generates the Dickson attributes, such as the required degree of the Dickson polynomial; the value of variable x, and calculates the public key.

**Algorithm 1: IoT device Registration**

*Input***:** Trusted Authority, Blockchain, IoT device.

*Output***:** Public key, Blockchain digital certificate, PUF Identity.

*Step 1*: The number of trusted authorities in a IoT network can be given as

13 $$\mathop \sum \limits_{i = 1}^{n} TA_{i} = \left\{ {TA_{1} , TA_{2} , \ldots ,TA_{n} } \right\}$$

*Step 2*: Each trusted authority is engulfed with several quantity of IoT devices given by

14 $$\mathop \sum \limits_{i = 1}^{n} I_{i} = \, \{ I_{1} ,I_{2} , \ldots ,I_{n} \} \to TA_{i}$$

*Step 3*: The trusted authority selects an two random large integers pk, y $\in$ $Z_{q}^{*}$.

*Step 4*: Compute

15 $$Dick_{Alice} = D_{pk} {\text{y}}$$

where $Dick_{Alice}$ is the Dickson polynomial.

*Step 3*: Therefore the public key generated for each IoT device can be defined as

16 $$({\text{x}},Dick_{Alice} ) \to I_{i}$$

*Step 4*: A physically unclonable identity is generated for each of the IoT device corresponding to a particular trusted authority.

17 $$\mathop \sum \limits_{i = 1}^{n} puf\,ID_{i } = \left\{ {puf\,ID_{1 } , puf\,ID_{2 } , \ldots , puf\,ID_{n } } \right\}$$

*Step 5*: In order to perform communication smart contracts are used. These smart contracts are generated by the blockchain technology in the form of a digital certificate called BDC.

*Step 6*: The blockchain digital certificate gets embedded with metadata like $pufID_{i }$, corresponding trusted authority identity, IoT device name and type which are of the form

18 $${\text{BDC}} \rightarrow \left\{ {pufID_{i } , TA_{i} , I_{i} } \right\}$$

### Group creation phase

In this phase one of the trusted authorities initiates the process of group creation. The trusted authority that initiates the process of group creation is called as guarantor TA. The guarantor TA is mainly responsible for generating the name and identity of a group.

**Algorithm 2: Group Creation**

*Input***:** Guarantor TA, Tie-group function, physical unclonable identity $pufID_{i }$.

*Output***:** Group name and Group Identity.

*Step 1*: The guarantor TA is mainly responsible for grouping the IoT devices to a particular trusted authority. In order to perform this, the guarantor TA utilizes tie-group smart contract function that utilizes XOR operation where the $pufID_{i }$ and the group identities are merged together.

19 $$TGF\left( x \right) = \left\{ {puf\,ID_{i } || group\,ID} \right\}$$

*Step 2*: Every smart contract contains the information about the group name, identity and the physical unclonable identities of all the corresponding IoT devices.

20 $${\text{Smart}}\;{\text{ Contract SC}} \to \left\{ {puf\,ID_{i } , group\; name, group\,ID} \right\}$$

*Step 3*: Finally the blockchain contains the public keys of all the participating IoT devices.

### Group authentication phase

Once the smart contract for each group gets created, the group authentication phase gets initiated. The group authentication phase involves two distinct phases namel`y secret sharing of Dickson degree and the smart contract execution.

#### Secret sharing of Dickson polynomial degree

**Figure 5:**
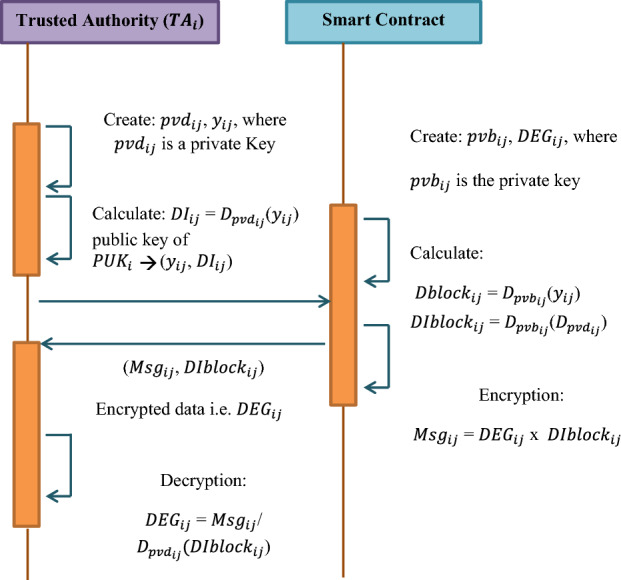
Secret sharing of dickson degree.

In order to facilitate data transmission a trusted handshake has to be performed between the IoT devices. For authentication, it is much essential to share the degree of the Dickson to be utilized in the Dickson polynomial. Each IoT device will be issued a distinct degree which is a large number used for the smart contract. Since these smart contracts are based on the blockchain technology the degree for each IoT device will be shared secretly. Hence in order to facilitate this, Dickson polynomial based public key cryptography is used as shown in Fig. 5.

**Algorithm 3: Secret Sharing**

*Input***:** Trusted Authority $TA_{i}$; Dickson polynomial Degree $DEG_{ij}$; IoT Devices $I_{i}$.

*Output***:** Smart contract makes degree to be shared.

*Step 1*: The trusted authority will generate a large number $pvd_{ij}$ and another number $y_{ij}$ where y $\in$ {− 1, 1}.

21 $$Step\; \, 2:{\text{ Calculate}}\;DI_{ij} \, = \,D_{{pvd_{ij} }} (y_{ij} );{\text{ where}}\;pvd_{ij} \; {\text{is}}\;{\text{ a}}\;{\text{ private}}\;{\text{ key}}$$

22 $$Step \, \;3:{\text{ The}}\;{\text{ public}}\;{\text{ key}}\;{\text{ of}}\;{\text{ each}}\;{\text{ trusted}}\;{\text{ authority }}\;{\text{will}}\;{\text{ be }}\;{\text{given}}\;{\text{ by}}\;PUK_{i} \, = \,\{ y_{ij} ,DI_{ij} \}$$

23 $$Step\; \, 4:{\text{The}}\;{\text{blockchain}}\;{\text{smart}}\;{\text{contract}}\;{\text{will}}\;{\text{generate}}\;{\text{a}}\;{\text{large}}\;{\text{number}}\;{\text{and}}\;{\text{a}}\;{\text{degree}}\;DEG_{ij}$$

where $pvb_{ij}$ is a private key of the blockchain.

24 $$Step\;5:{\text{Smart}}\;{\text{contract}}\;{\text{will}}\;{\text{calculate}} \;Dblock_{ij} \, = \,D_{{pvb_{ij} }} (y_{ij} ); DIblock_{ij} \, = \,D_{{pvb_{ij} }} (D_{{pvd_{ij} }} )$$

*Step 6*: Smart contract will then bind the degree of the dickson polynomial to that of the IoT device and is stored in the blockchain.

*Step 7*: Smart contract will then performs encryption by embedding the degree to that of the message by calculating

25 $$Msg_{ij} \, = \,DEG_{ij} \times DIblock_{ij}$$

*Step 8*: Smart contract then sends the message to the corresponding IoT device.

*Step 9*: IoT device then performs decryption by debinding the degree $DEG_{ij}$ from the message $Msg_{ij}$ by multiplying it with $D_{{pvd_{ij} }} (DIblock_{ij}$). Which is a Dickson value calculated using the IoT device’s private key and $(DIblock_{ij}$).

[Note: This is highly successful due to the semigroup property of the Dickson polynomial].

The Eq. (26) represents the first phase of the group authentication scheme.

26 $$\begin{aligned} DIblock_{ij} & = D_{{pvb_{ij} }} (DI_{ij} ) \\ & = D_{{pvd_{ij} }} \left( {DIblock_{ij} } \right) \\ & = D_{{pvd_{ij} }} (D_{{pvb_{ij} }} (y_{ij} )) \\ & = D_{{pvb_{ij} }} (D_{{pvd_{ij} }} \left( {y_{ij} } \right)) \\ \end{aligned}$$

Smart contract performs the secret sharing methods for each of the individual IoT device associated with various trusted authorities by associating the computed degree $DEG_{ij}$. Therefore each distinct IoT device will have its own degree of the dickson polynomial.

#### Execution of smart contract

Upon message transmission amongst IoT devices pertaining to a group, smart contract gets validated by examining with the group identity. Upon successful validation and verification, the IoT devices in a group proceeds through the group authentication. The proposed authentication scheme follows the authentication algorithm by executing the smart contract and performs group authentication.

**Algorithm 4:**
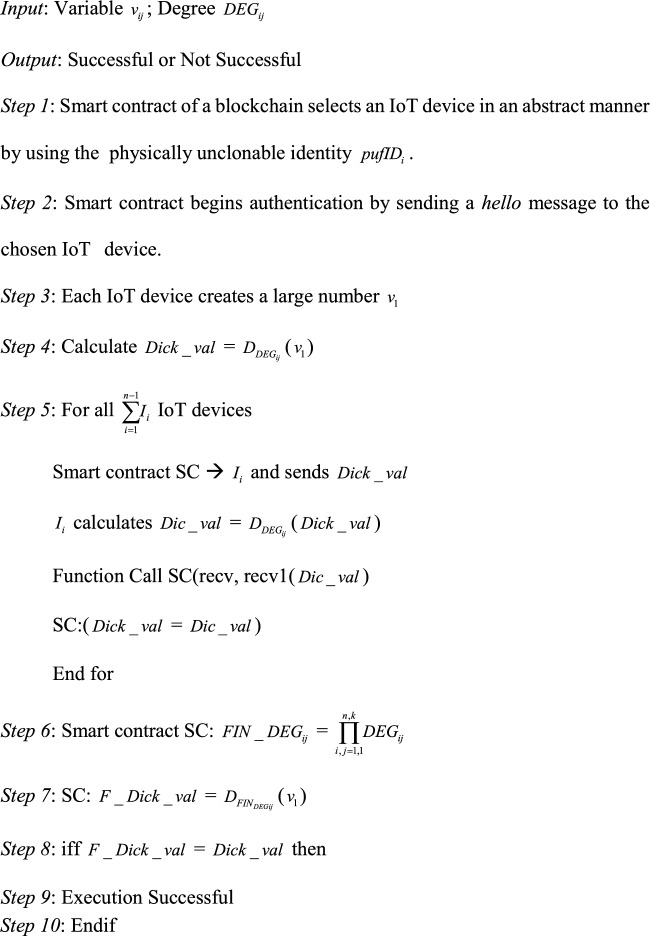
Execution of Smart Contract

To perform group authentication initially an IoT device is chosen by the smart contract by using the physically unclonable identity. In order to initiate the conversation, *hello* message is sent. The corresponding device calculates the $Dick\_val$ = $D_{{DEG_{ij} }}$($v_{1}$). In order to perform authentication this value is then sent to the smart contract. After receiving this value the smart contract chooses one of the other IoT devices and sends it to the *recv* function*.* This function is then merged to catch the new $Dic\_val$ which was sent to the IoT device generated by the blockchain. After the new value gets received the degree of the IoT device can be computed as $Dic\_val$ = $D_{{DEG_{ij} }}$($Dick\_val$). The older value is then assigned to the new value by using the smart contract. The value that is received by the last IoT device in the group is then stored for all the remaining devices by using the smart contract. From step 6 the number of trusted authorities can be defined as n and the number of IoT devices in the group be given by k. When the final value of the last IoT device is equal to that of the initial value then it implies that all the IoT devices shared the same $DEG_{ij}$ by using the shared smart contract. Since method of approximation is utilized for calculate the *Dick_val* the values received will be of same value but a slight difference can be observed. When the smart contract gets executed it implies that group authentication has become successful. When an intruder performs physical or tamper-proof attacks, the final dick value will never be equal to that of the first value. IF in case any of the participating IoT devices gets compromised/replaced/added/removed by any other IoT device group authentication has to be performed again.

## Security analysis

Security Analysis of our proposed dickson polynomial based secure group authentication scheme has been carried by two ways namely formal and informal security analysis.

### Formal security verification using AVISPA tool

**Figure 6:**
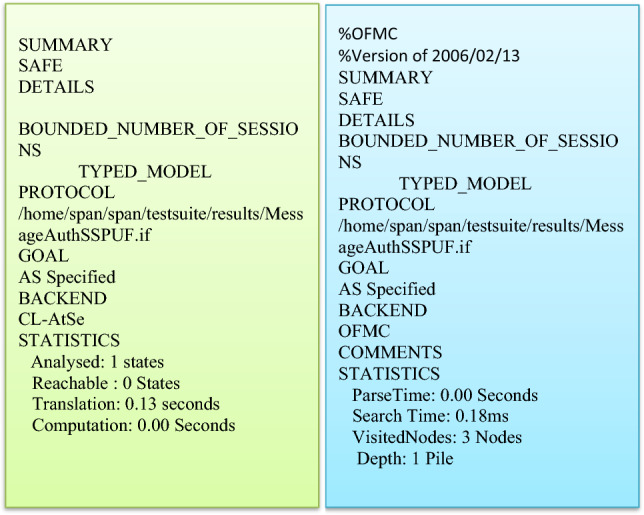
Results of Simulation using AVISPA tool.

This section highlights the evaluation process carried out for the formal security verification by utilizing Automated Validation of Internet Security Protocols and Applications (AVISPA) tool. The tool consists of back-ends and absorption roles coded in High Level Protocol Specification Language (HSPSL). The backends of AVISPA tool gets elaborated in^53,54^. It is apparent that the proposed scheme is SAFE under the backends OFMC and CL-AtSe as shown in Fig. 6. Thus the proposed secure group authentication scheme is highly resilient towards replay and man-in-the-middle attacks.

### Formal security analysis

The proposed scheme has been validated formally by using Burrows-Abadi-Needham (BAN) logic^54^. The rules to be followed for BAN logic are as follows:

- $R_{1}$: Message-Meaning Rule: $$\frac{{Q| \equiv Q\begin{array}{*{20}c} {SK} \\ \leftrightarrow \\ \end{array} R,~Q \triangleleft \left\langle I \right\rangle _{{SK}} }}{{Q\left| { \equiv BS} \right| \sim I}}$$
- $$R_{2}$$: Jurisdiction Rule: $$\frac{{Q\left| { \equiv BS \Rightarrow I, Q} \right| \equiv BS \Rightarrow I}}{Q| \equiv I}$$
- $R_{3}$: Session Key Rule: $$\frac{{Q\left| { \equiv \# I, Q} \right| \equiv R| \equiv I}}{{Q| \equiv Q\begin{array}{*{20}c} {SK} \\ \leftrightarrow \\ \end{array} R}}$$

Here Q and BS (blockchain server or the smart contract) are the communicating agents, I is the statement and SK is the secret key. The premises and the symbols utilized for the BAN logic are depicted in the Table 1

**Table 1:** BAN logic formulas.

Symbol	Description
$Q| \equiv I$, *Q* $\triangleleft$ *I*	Q believes I, Q receives I
$Q| \equiv \sim I$, $Q \Rightarrow I$	Q once sent I, Q has full control over I
#(I),$I_{SK}$	I is fresh, I is combined with SK
$$\left\{ I \right\}_{SK} , Q| \equiv Q\begin{array}{*{20}c} {SK} \\ \leftrightarrow \\ \end{array} BS$$	I is encrypted with SK, I believes that SK is shared between Q and BS

The BAN Logic objectives to be proved are;

- Objective 1: TA $$| \equiv pufID_{i}$$,$${\text{x}},{ }Dick_{Alice}$$, $$Msg_{ij}$$
- Objective 2: SC |$$\equiv groupID_{i} , x, BDC$$
- Objective 3: $$I_{i} \left| { \equiv Dick\_val} \right| \equiv$$ $$DEG_{ij}$$
- Objective 4: $$I_{j} \left| { \equiv I_{i} } \right| \equiv groupID_{i} ,Msg_{ij} ,DEG_{ij}$$

In order to carry out the formal analysis, the message transmission involves the IoT device, Trusted Authority and the Smart contract of the blockchain.

- $$M_{1}$$: $$TA \to SC$$: {$$pufID_{i}$$,$${\text{x}},{ }Dick_{Alice}$$, $$Msg_{ij}$$}
- $$M_{2} :SC \to TA:\left\{ {groupID_{i} , x, BDC} \right\}$$
- $$M_{3} :TA \to I_{i} :Dick\_Val$$
- $$M_{4} : I_{i} \to I_{j} :\left\{ {groupID_{i} ,Msg_{ij} ,DEG_{ij} } \right\}$$

For our proposed scheme the BAN logic assumptions are as follows:

- $$A_{1} : I_{i} | \equiv I_{i} \begin{array}{*{20}c} {\left( {x, Dick_{Alice} } \right)} \\ \leftrightarrow \\ \end{array} TA$$
- $$A_{2} :TA| \equiv SC \Rightarrow pufID_{i} , {\text{x}},{ }Dick_{Alice} ,Msg_{ij}$$
- $$A_{3} : TA| \equiv I_{i} \Rightarrow \left( {pufID_{i} , x} \right)$$
- $$A_{4} : SC| \equiv SC\begin{array}{*{20}c} {BDC} \\ \leftrightarrow \\ \end{array} TA$$
- $$A_{5} :I_{i} \left| { \equiv TA} \right| \equiv DEG_{ij}$$
- $$A_{6} :I_{i} | \equiv I_{i} \begin{array}{*{20}c} {DEG_{ij} } \\ \leftrightarrow \\ \end{array} I_{j}$$

The formal security proof for our proposed secure group authentication scheme can be defined as:

By message 2, the following statement is achieved:

- S1: SC $$\triangleleft \left\{ {groupID_{i} , x, BDC} \right\}_{{\left( {{\text{x}},{ }Dick_{Alice} } \right)}}$$

According to s1, A1 and R1, we have:

- S2: $$SC\left| { \equiv TA} \right| \sim groupID_{i} , x, BDC$$

According to S2, A1, R2, we have:

- S3:$$SC\left| { \equiv TA} \right| \equiv groupID_{i} , x, BDC$$

According to S3, A1, A4, R2, we have:

- S4: $$SC| \equiv groupID_{i} , x, BDC\quad \quad \quad \quad \left( {\text{Objective 2}} \right)$$

By message 1, the following statements are satisfied:

- S5: $$TA\left| { \equiv SC} \right| \sim pufID_{i}$$,$${\text{x}},{ }Dick_{Alice}$$, $$Msg_{ij}$$

According to S5, A2, A3, R2, we have:

- S6: $$TA\left| { \equiv SC} \right| \equiv pufID_{i}$$,$${\text{x}},{ }Dick_{Alice}$$, $$Msg_{ij}$$

According to S6, A2, A3, R3, we have:

- S7: $$TA| \equiv pufID_{i}$$,$${\text{x}},{ }Dick_{Alice}$$, $$Msg_{ij} \quad \quad \quad \left( {\text{Objective 1}} \right)$$

By message 3,

- S8: $$I_{i} \triangleleft D_{{pvb_{ij} }}$$($$D_{{pvd_{ij} }}$$)

According to S8, A5, R3, we have:

- S9: $$I_{i} \left| { \equiv TA} \right| \sim D_{{pvb_{ij} }}$$($$D_{{pvd_{ij} }}$$)

According to S9, A5, R3, we have:

- S10: $$I_{i} \left| { \equiv TA} \right| \equiv D_{{pvb_{ij} }}$$($$D_{{pvd_{ij} }}$$)

According to S10, A5, R2, we have:

- S11: $$I_{i} | \equiv DEG_{ij} \quad \quad \quad \quad \left( {\text{Objective 3}} \right)$$

By message 4,

- S12: $$I_{j} \triangleleft groupID_{i} ,Msg_{ij} ,DEG_{ij}$$

According to S12, A6, R1, we have:

- S13: $$I_{j} \left| { \equiv I_{i} } \right| \sim groupID_{i} ,Msg_{ij} ,DEG_{ij}$$

According to S13, A4, A5, A6, the following equations can be obtained as

- S14: $$I_{j} \left| { \equiv I_{i} } \right| \equiv groupID_{i} ,Msg_{ij} ,DEG_{ij} \quad \quad \quad \left( {\text{Objective 4}} \right)$$

The proposed secure group authentication scheme achieves all the objectives thereby gurantees mutual authentication between the IoT devices. By exchanging the dickson’s degree and when the value of the final dickson value is equal to that of the initially assigned value then the authentication becomes successful.

### Informal security analysis

Informal security analysis has been carried out in order to assess the security resilience under various attacks for our proposed authentication scheme. This informal security analysis carried out follows the same pattern as in^55^.

(i) *Data Integrity and Authentication* An IoT device must be authenticated prior to message transmission. Therefore, the sender a secret value $DI_{ij}$ = $D_{{pvd_{ij} }}$($y_{ij}$) used to generate a public key. The receiver performs validation by computing $DEG_{ij}$. Only when both the sender private key matches with the receiver’s key then the data is trustworthy and is highly confidential. Thus our proposed scheme achieves authentication by using the secret key and with the dickson’s degree.
(ii) *Mutual Authentication* In our proposed scheme, only the registered IoT device $I_{i}$ performs communication iff there is a secret key $D_{{pvd_{ij} }}$ and *groupID*. The smart contract in the blockchain gets executed by using $DIblock_{ij}$. On the other hand only the smart contract possesses its own blockchain secret key $D_{{pvb_{ij} }}$ so that the message can be encrypted. Therefore the message gets decrypted $DEG_{ij}$ = $Msg_{ij}$/ $D_{{pvd_{ij} }} (DIblock_{ij}$). Hence Mutual authentication is achieved and it cannot be attacked.
(iii) *Session Key Agreement* In our proposed scheme the message transmission happens only when the IoT devices are authenticated by using the smart contract secret key $ck\_val$ ; where $Dick\_val$ = $D_{{DEG_{ij} }}$($v_{1}$). Hence our proposed scheme is able to achieve session key agreement.
(iv) *Privacy of the IoT devices* In our proposed scheme, for each session a device needs to provide a legal *pufID* that cannot be utilized again. It is because each time to perform a transaction the identity of the IoT device is binded with the group identity which can only be validated by the blockchain. Due to the synchronization loss, the IoT device used one of the other identities from $\mathop \sum \limits_{i = 1}^{n} pufID_{i } .$ Following that the device erases its identity from its memory. This technique achieves traceability and provides ability to be resilient against eavesdropping attack.
(v) *Replay attack* The proposed authentication scheme is utilized to perform group authentication between when communication happens or message transmission is about to be made. When an authentication request has been made, all IoT devices in a group and the blockchain create the required parameters and keys like $pvd_{ij}$,$y_{ij}$,$pvb_{ij}$,${ }DEG_{ij}$. The communication is always accomplished by the use of a timestamp. Since the creation of the required parameters values and variables happens only when an authentication request is made the proposed group authentication scheme is highly resilient against replay attacks.
(vi) *Man-in-the-middle attack* Whenever the IoT device pertaining to a group wants to communicate with the other it has to perform a trusted handshake which has to be approved by the execution of smart contract. All the IoT devices and the blockchain uses a secret called Dickson degree ($DEG_{ij}$) for one single communication. This information is shared as secret and hence it differs for each of the distinct IoT device. Therefore it is impossible for any node inside the network can able to crack the secret. The authentication is performed by sharing another secret value (*Dick_v1)* between the IoT devices and the smart contract of the blockchain. The authentication gets acknowledged and verified by the blockchain only when all the participating IoT devices in a group calculate their corresponding *Dick_v1*. Whenever an intruder snatches into a communication line it is impossible to revive an authentication request one more time for the same IoT device. It is because the blockchain will create a new secured secret ($DEG_{ij}$) using *pufID*_*ij.*_ of the listed IoT device only. Therefore it impossible to perform a man-in-the-middle attack by acquiring the initial shared secret $DEG_{ij}$ between the blockchain and the corresponding IoT device since another authentication request is needed.
(vii) *Offline password guessing attacks* The offline guessing of the secret keys and the variables will not help the attackers to gain access to the stored secrets in the blockchain. Hence it is not possible to crack the secrets even if an authentication request is made. Also even if the secret variables of one of the IoT device get retrieved; it is impossible to crack the authentication of other devices and the same device. Thus it is computationally hard and resilient against offline guessing attacks.
(viii) *Smart card stolen and Impersonation attacks* The proposed secure authentication scheme utilizes a distinct physically unclonable identity *pufID*_*ij*_ for each individual IoT devices. All the communication is supposed to be verified by using the physically unclonable identity. During the first phase of group authentication the trusted authority utilizes the metadata *pufID*_*ij*_ and other parameters in order to create a blockchain digital certificate BDC. The certificate has to be verified for every transaction. It is impossible for the intruder to crack the communication or gain access to the IoT device since the digital certificate is stored in both the blockchain and the IoT device. Hence it impossible to perform impersonation attacks. Also it is impossible for the compromised or the stolen IoT device for making authentication requests, since the verification of the digital certificate of a corresponding IoT device has to be verified in the blockchain. It is easy to identify this information since the blockchain operates in the form of last-in-first order which information reveals that the corresponding IoT device is no more valid upon validation. Hence it is computationally hard to crack for stolen smart card attacks.
(ix) *Ephemeral secret key leakage attack* The proposed authentication scheme is secure against ephemeral secret key leakage attacks which are mainly dependent on the keys based on the timestamp. The keys like $pvd_{ij}$,$y_{ij}$,$pvb_{ij}$ are generated based on the authentication requests made. The short-term keys like $DEG_{ij}$ and $Dick\_val$ are created afresh upon the request for authentication. Long-term keys are mainly created in order to ensure the secrecy of the transaction. Even if the short-term keys gets compromised for a session it is computationally hard to crack and it does not have any impact over previous or future authentication requests. Because of this approach both forward and backward secrecy can be achieved. This makes impossible even if a compromisation is made for one session due to the use of dickson polynomial it is hard to crack. Since the overall value of the blockchain should be the same as that of distinct IoT devices successful authentication can be made.

## Performance analysis

The proposed dickson polynomial based secure group authentication scheme has been implemented by using the Ethereum blockchain. Python is the programming language used and smart contract was executed by using Solidity. Ethereum’s Goerli and Sepolia testnets are utilized to assess the performance of a smart contract. Goerli’s testnet is a public network and uses the proof-of-stake consensus algorithm. The size of this blockchain is similar to that of the Ethereum mainnet^56^. The hardware utilized for the implementation is an AMD Ryzen 7 8500H with Radeon graphics processor, with a RAM of size 8 GB and a dedicated hard disk of 100 GB operating in virtual machine running on Linux Ubuntu 18.04.6 LTS OS.

**Figure 7:**
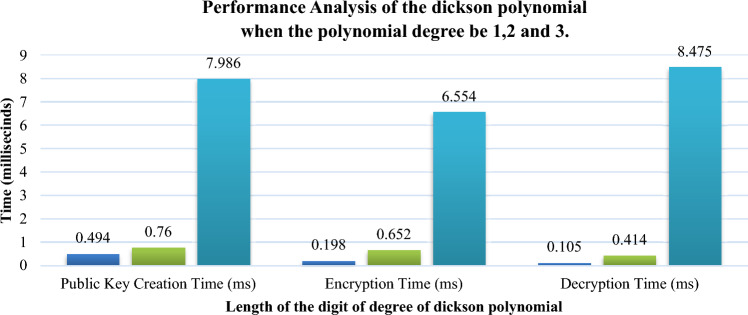
Performance analysis of the dickson polynomial degree of 1, 2, and 3.

Dickson polynomial value can be computed by two methods namely recursive and approximation. In case of recursive computation, the dickson polynomial exhibit high computation cost beyond a certain degree. For our proposed scheme approximation method is used. Though the values obtained under both these methods may differ it was actually nearby to that of the recursive strategy. In order to understand the execution of the scheme time analysis has been carried for various process namely public key creation, encrypting and decrypting the degree variable. Experiments have conducted for various digital values of the degree, time taken by the random number generator is included in the time analysis. Digit lengths from 1 to 3 have been used which can be identified from the Fig. 7.

**Figure 8:**
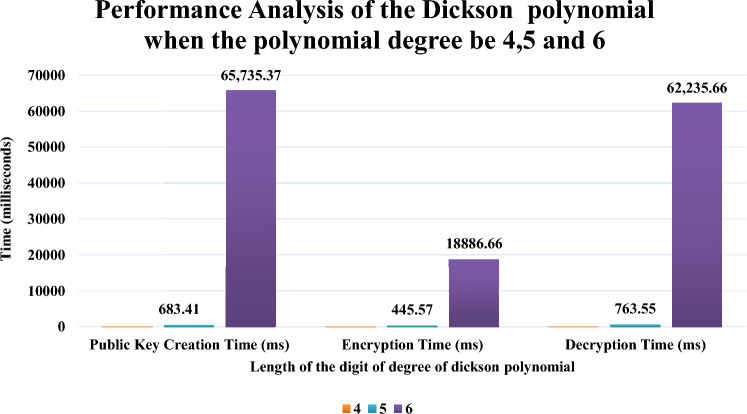
Performance analysis of the dickson polynomial degree of 4, 5, and 6.

It is apparent that time necessary to create a public key and to perform encryption and decryption increases with increase in the length of the digits. If the length of the digit is 1, it is a polynomial degree corresponding to a single bit which can be described as a linear equation. From the graph it is observed that the time required to public key creation, encryption and decryption are 0.494 ms, 0.198 ms and 0.105 ms. If the length of the digit is 2, (i.e. double degree polynomial) the time taken gets observed as 0.760 ms, 0.652 ms and 0.414 ms. When the length of the digit is 3, the time taken gets observed as 7.986 ms, 6.554 ms and 8.475 ms. Figure 8 projects the performance aspects in the graph when the length of the digits become 4,5 and 6. From the graph it is apparent that when the length of the digit increases, time increases in an exponent order. For the creation of public key the time for digit length 4 will rise from 55.64 to 65,735.37 ms. In case of encryption, time increases from 94.88 to 18,886.60 ms when the length of the digit is 6. In case of decryption the time increases from 50.22 ms for when the length of the digit is 4 to 62,235.66 ms when the digit length increases to 6. For larger digit lengths it would take high exponential time where group authentication is not possible within time. Ethereum’s Goerli Testnet has the used for deploying the smart contract by using the blockchain related parameters for our dickson polynomial based secure group authentication scheme. Table 2 describes the performance aspects of the blockchain technology. Though permissioned private blockchain can be utilized the proposed authentication scheme used one blockchain node. Table 2 provides a clear cut understanding about the cost and performance when the trusted authorities utilize ethereum’s mainnet with real ethers. For smart contract deployment the compute units are 1.4 and for performing the group authentication the compute units are 4.1 while group authentication. Response time of the median for the distribution of the smart contract was 15 ms and 21 ms. It is observed that the gas price during the deployment was 96,780 Wei and 94,450 Wei while performing group authentication. The estimated gas during the deployment is 354,129 Wei and 20,153 Wei. Ether_getBlockByNumber is a function that hunts for a block and charges ethers for the base fee and the gas utilization.

**Table 2:** Performance analysis for Blockchain utilized.

Parameters/use cases	Distribution	Transaction for group authentication
Avg Computing units	1.4	4.1
Median Response (ms)	15 ms	21 ms
Ether_Gasprice	96,780	94,450
Ether_EstimateGas	354,129	20,153
Ether_getBlockByNumber: Base fee per gas	89,351	105,396
Ether_getBlockByNumber: Gas Used	25,128,637	17,079,755
Maximum Fee Per Gas	–	1,300,115,522
Maximum Priority Fee Per Gas	–	1,300,000,000
Ether_getTransactionReceipt: Cumulative Gas used	14,528,560	–
Ether_getTransactionReceipt: Effective Gas used	96,780	–
Ether_getTransactionReceipt: Gas used	354,129	–

For the exploitation of the smart contract, the base fee and the gas used will be 89,351 Wei and 25,128,637 Wei and for group authentication it will be 105,396 Wei and 17,079,755 Wei. Fee Per Gas is defined by the absolute maximum gas price when a user wants to insert a block in a blockchain. Therefore the maximum gas price paid by the trusted authorities for performing group authentication was 1,300,115,522 Wei. Maximum priority fee per gas is the price of the maximum gas set by the user that will be paid to the miners for performing block insertion and ir accounts to 1,300,000,000 Wei. The transactions receipt are supposed to analyze and to understand the cost for transaction processing during the execution of the smart contract. Total gas consumption in a given block, including that for deployment and subsequent transactions, was calculated to be 14,528,560 Wei. The smart contract deployment was observed to deduct 96,780 Wei per gas, from the TA account. In addition, the gas utilized to deploy the smart contract was confirmed to be 354,129 Wei, which is the same amount as the gas estimated by the Ether_EstimateGas function.

The proposed dickson polynomial based secure group authentication scheme utilizing blockchain technology has been analyzed for throughput and transaction latency. The major aim of using blockchain makes the participants to submit the transactions followed by verification and ordering. This leads to generation of blocks where the results of the transactions are stored. According to Hyperledger Performance and Scale Working Group^57^ several performance metrics has been proposed in order to evaluate the performance of the blockchain. The following are the performance metrics namely **1. Transaction throughput:** It can be defined as summation of all the transactions that are successfully committed within the given time period usually seconds. **2. Transaction Latency:** It can be defined as the time taken to store a transaction in the hyperledger fabric. The results are compared with the benchmarks take from the related works. The checking has been done by using the hyperledger caliper^58–60^ with which the administrator has to configure the blockchain.

**Figure 9:**
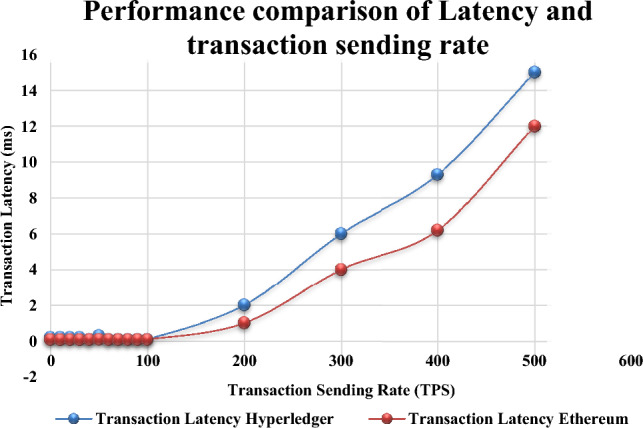
Performance Comparison of Latency and Transaction Sending Rate.

**Figure 10:**
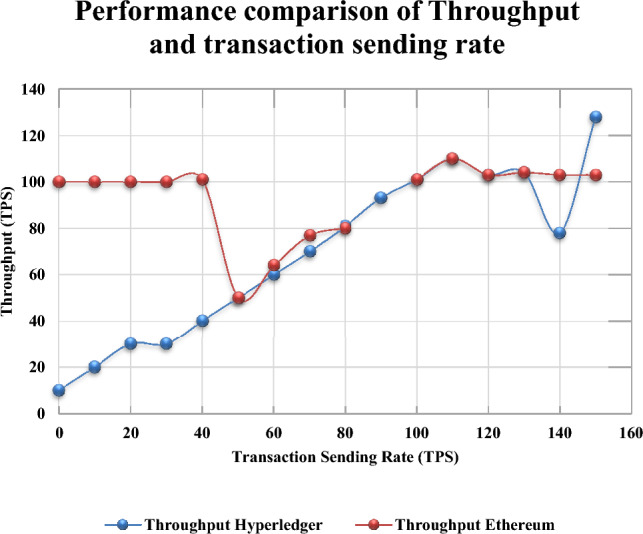
Performance Comparison of Throughput and Transaction Sending Rate.

In our proposed group authentication scheme, the latency can be defined as the time taken by the patron trusted authority for verifying the new blocks. Block size determines the latency and throughput^61,62^. Transaction latency can be defined as the time taken by the system to attain consensus. The latency usually happens when new block validations are detected after the node gets started. The analysis has been preceded by a set of transactions namely open, exchange and query. Latency can be met when the resources are allocated for the blockchain network. The transactions are sent from 20 to 500 Transactions Per Second (TPS). In order to assess the performance of each benchmarks 1000 transactions are performed to evaluate the maximum, average and minimum latency and throughput. Figure 9 depicts the transaction latency for each round. One second is the minimum latency and the maximum will be 100 TPS. A significant drop happened when the transactions are sent at a rate of 120 TPS, which is the highest sending rate for blockchain system under test. Figure 10 represents the transaction throughput for distinct transaction sending rate. Thus it is observed that the Ethereum based blockchain has outperformed the hyperledger fabric where the maximum latency results to 12 s as the number of transactions. At the beginning no high loads can be impulse on the group of IoT devices since the maximum latency remains constant^63^. Also the blockchain size, configuration, number of channels, ordering service, users and endorsing nodes affects the latency.

**Figure 11:**
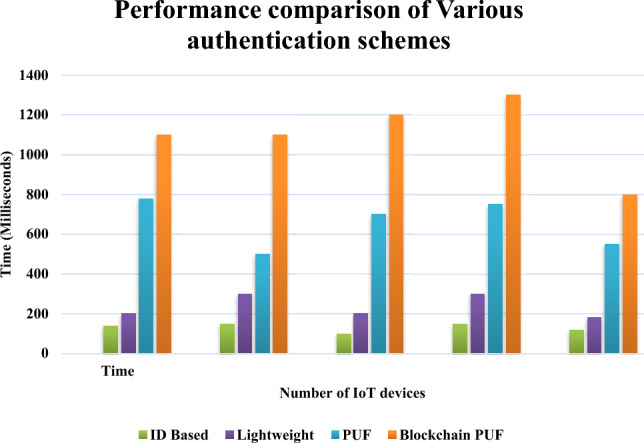
Performance comparison of various authentication schemes along with Dickson polynomial based secure group authentication scheme.

Figure 11 depicts the performance comparison of various schemes to that of our PUF-based secure group authentication scheme using blockchain technology.

## Conclusion

Security and trust management is very vital and hard in case of Internet of Things environment when they are controlled by a various entities. The proposed secure group authentication scheme utilizes dickson polynomial and blockchain technology. The proposed framework possess the ability to authenticate IoT devices located on various entities and to permit safe data transfer between these communities. Python was used to develop the proposed framework for analysing the dickson polynomial's temporal complexity, and the resulting Solidity-based smart contract was deployed on Ethereum's Goerli testnet. The proposed group authentication scheme has been used in order to analyze different parameters of blockchain namely effective gas used, estimated gas, base fee per gas and gas.

Blockchain based solutions suffers from a serious problem named scalability. This can be tested in the future. The Goerli network, a massive simulation of the Blockchain with nearly the same scalability parameter as the real Blockchain, was used to evaluate the framework in this study. Ideas are needed to speed up the Blockchain's response time for real-time group authentication. The proposed scheme can be revived in the near future to make dickson polynomial less computation sonerous for the smart contract. The suggested system can be altered to shift the burden of big complex polynomial approximations from smart contracts to a reliable third party. It will be fascinating to examine how the new mechanism affects the various performance measures.

## Data Availability

The datasets used and/or analysed during the current study available from the corresponding author on reasonable request.

## Abbreviations

IoT: Internet of Things
TA: Trusted authority
PUF: Physically unclonable function
SC: Smart contract
ms: Milliseconds
$pufID_{i }$: Physically unclonable function identity of an IoT device
$I_{i }$: Ith entity of a IoT device
$TA_{i }$: Ith entity of a trusted authority
$D_{n }$: Dickson polynomial of nth degree
$pka \left( x \right)$: Private key of alice
$pkb \left( x \right)$: Private key of Bob
$D_{pk}$: Private key embedded with a dickson polynomial
$Dick_{Alice}$: Public key of Alice of a dickson polynomial
BDC: Blockchain digital certificate
$TGF$: Tie-group Function
groupID: Identity of a group of IoT device
$pvd_{ij} ,y_{ij}$: Private key, large number for one session between ith and jth device
$DI_{ij}$: Dickson public key of an IoT device
$PUK_{i}$: Public key between ith and jth IoT group
$Dblock_{ij}$: Dickson value computed by a smart contract using $DIblock_{ij}$
$pvb_{ij}$: Public key, for one session between ith and jth device
$Msg_{ij}$: Message to be sent between ith and jth device
$DIblock_{ij}$: Dickson vaue by a smart contract using $DI_{ij}$
$D_{{pvb_{ij} }}$: Dickson private key of the blockchain
$D_{{pvd_{ij} }}$: Dickson private key of the IoT device
$DEG_{ij}$: Degree of a dickson polynomial
$Dick\_val$: Dickson value of an IoT device
